# APOL1 risk variants and kidney disease: what we know so far

**DOI:** 10.1590/2175-8239-JBN-2017-0033

**Published:** 2018-07-26

**Authors:** Tobias August Siemens, Miguel Carlos Riella, Thyago Proença de Moraes, Cristian Vidal Riella

**Affiliations:** 1 Hospital Universitário Cajuru, Curitiba, PR, Brasil.; 2 Fundação Pró-Renal, Curitiba, PR, Brasil.; 3 Pontificia Universidade Católica do Paraná, Departamento de Medicina, Curitiba, PR, Brasil.; 4 Beth Israel Deaconess Medical Center, Boston, MA, United States.

**Keywords:** Genetics;Apolipoprotein L1, Kidney, Glomerulosclerosis, Focal Segmental, AIDS-Associated Nephropathy, Nephrosclerosis, Renal Insufficiency, Chronic, Genética, Apolipoproteína L1, Rim, Glomerulosclerose Segmentar e Focal, Nefropatia Associada a AIDS, Nefroesclerose, Insuficiência Renal Crônica

## Abstract

There are striking differences in chronic kidney disease between Caucasians and African descendants. It was widely accepted that this occurred due to socioeconomic factors, but recent studies show that apolipoprotein L-1 (*APOL1*) gene variants are strongly associated with focal segmental glomerulosclerosis, HIV-associated nephropathy, hypertensive nephrosclerosis, and lupus nephritis in the African American population. These variants made their way to South America trough intercontinental slave traffic and conferred an evolutionary advantage to the carries by protecting against forms of trypanosomiasis, but at the expense of an increased risk of kidney disease. The effect of the variants does not seem to be related to their serum concentration, but rather to local action on the podocytes. Risk variants are also important in renal transplantation, since grafts from donors with risk variants present worse survival.

## RESUMO

Existem importantes diferenças na doença renal crônica entre caucasianos e afrodescendentes. Foi amplamente aceito que isso ocorreu devido a fatores socioeconômicos, mas estudos recentes mostraram que as variantes gênicas da apolipoproteína L-1 (APOL1) estão fortemente associadas à glomeruloesclerose segmentar e focal, nefropatia associada ao HIV, nefroesclerose hipertensiva e nefrite lúpica na população afrodescendente. Essas variantes chegaram à América do Sul através do tráfico intercontinental de escravos, e proporcionaram uma vantagem evolutiva aos portadores, protegendo contra formas de tripanossomíase, mas à custa de um maior risco de doença renal. O efeito das variantes não parece estar relacionado à sua concentração sérica, mas sim à sua ação local sobre os podócitos. Variantes de risco também são importantes no transplante renal, já que enxertos de doadores com variantes de risco apresentam pior sobrevida.

## RACIAL DIFFERENCES IN CHRONIC KIDNEY DISEASE

According to the United Nations (UN), chronic kidney disease (CKD) is a global health problem. CKD mortality almost doubled between 1990 and 2010, and by the end of 2013, over three million people were undergoing renal replacement therapy (RRT) worldwide, two and a half million were on hemodialysis or peritoneal dialysis, and close to 700,000 had received a kidney transplant. These numbers are predicted to continue to rise as the worldwide prevalence of CKD increases at a rate of 6% per year.^1^

There are striking differences in CKD incidence when stratified by race. African Americans (AA) have four times higher incidence of CKD than Caucasians.^1^^,^ 2 Moreover, AA manifest a more progressive course of the disease.^3^ This higher incidence of CKD in AA was first observed thirty years ago^4^^,^ 5 and in 2004, the incidence of CKD in AA was 1000 per million people in the United States (US), compared to only 260 per million people in Caucasians.^4^ Several hypothesis have emerged to try to explain this disparity. One hypothesis was the higher proportion of AA living at lower socioeconomic status, with poor access to health care. This would have led to late diagnosis and treatment of diseases such as diabetes *mellitus* (DM) and hypertension (HTN), two major causes of CKD.^6^ It has also been hypothesized that the ethnic differences were justified by lack of affordable healthy foods or to exposure to environmental toxic factors.^4^ In 1988, Brenner proposed that AA presented fewer glomeruli at birth, which would increase susceptibility to CKD in adulthood.^7^ In addition, he proposed that this mechanism could explain the increased prevalence of diabetic nephropathy in this population. However, further studies did not confirm this hypothesis.^8^

This review article addresses another hypothesis for this racial disparity, which has been gaining strength in recent years in the scientific community: the mutations in the APOL1 gene, which would be related to a higher prevalence of certain nephropathies in Afro-descendants. The article also reviews how these mutations were discovered and what are their possible origin, existing prevalence studies on the subject, the theories that explain the toxicity of the mutation and its effects on podocytes, and finally the relationship between APOL1 mutations and cardiovascular risk as well as their implications in transplantation.

## DISCOVERY OF THE APOL1 POLYMORPHYSMS AND ASSOCIATION WITH CKD

The advent of Next Generation Sequencing led to cheaper and faster DNA sequencing, which in turn catalyzed a leap in the field of genetics research. The new technology allowed large-scale studies focusing on single nucleotide polymorphisms (SNPs) and their association with disease. It used mapping by admixture linkage disequilibrium (MALD) technique /genome wide admixture mapping to quantify the ancestry percentage of each locus in a gene.^9^ Then, genomic regions were identified, where AA patients with CKD had increased African ancestry, compared with healthy controls.^10^ The analysis led to a specific region on chromosome 22q12 with more than 21 genes, including the gene *MYH9*. This gene encodes a protein expressed in podocytes that is essential to the proper functioning of its cytoskeleton and intercellular adhesion. This physiological plausibility led researches to suggest that the *MYH9* gene was the culprit for the increased susceptibility of AA to non-diabetic nephropathy and focus segmental glomerulosclerosis (FSGS).^11^^,^ 12

Despite substantial effort by the scientific community, including detailed genotyping and sequencing of the *MYH9* gene, a mutation related to a deleterious effect on kidney function was not found.^1^ The focus then shifted to neighboring genes on chromosome 22. Genovese et al. compared 205 AA patients with biopsy-proven FSGS with 180 AA subjects without kidney disease and reanalyzed the 22q12 chromosome region, using data from the International HapMap Project and the 1000 Genome Project.^13^^,^ 14 Approximately twenty kilobases away from the *MYH9* gene, the *APOL1* gene was located, with the presence of mutations with strong statistical power associated with CKD in AA^15^. A total of 7479 SNP changes were found, three of them statistically related to increased risk of CKD in AA. Two of them (rs73885319 and rs60910145) are situated on the last exon of the *APOL1* gene (exon seven) and are the result of two amino acid substitutions, serine for glycine and isoleucine for methionine, at positions 342 and 384, respectively. These two mutations are now referred to as G1 allele, because they are in complete linkage disequilibrium (r^2^ = 1.0) with each other (i.e., they are always present together within an allele). The third mutation found (rs71785313), also on the exon seven of the gene *APOL1*, just twelve base pairs away from the G1 mutation site^16^, is a deletion of two amino acids (asparagine and tyrosine amino acid positions 388 and 389) and is called G2 allele. The two mutations are in perfect negative linkage disequilibrium (i.e, they never occur on the same allele).^17^ Two conclusions can be drawn from the described features: (a) G1 and G2 alleles have appeared in nature independently; (b) the two alleles have never undergone genetic recombination (since in perfect negative linkage disequilibrium). Therefore, there is no haplotype carrying G1 and G2 simultaneously.

The presence of variants of the APOL1 gene is possible in the homozygous (G1/G1 or G2/G2), compound heterozygote (G1/G2) or heterozygote (G1/G0 or G2/G0) forms. Patients present an increased risk of nephropathy only in homozygous and compound heterozygote genotypes (presence of two risk alleles); therefore, these forms were called high risk genotype (HRG). The heterozygous form, with the presence of only one risk allele, was called low risk genotype, since it did not increase the risk of kidney disease in patients.

Several subsequent studies have confirmed these findings (Table 1). AA carriers of the HRG had approximately three times greater risk of developing lupus nephritis, seven times greater risk of developing hypertensive nephrosclerosis, 17 times greater risk of developing primary FSGS, 29 times greater risk to develop HIV-associated nephropathy (HIVAN) when compared to non-AA controls^17^^,^ 18 and an association between the HRG and increased risk of sickle cell nephropathy^19^. Furthermore, the presence of the HRG accelerated the progression of CKD in cases of FSGS and hypertensive nephrosclerosis despite immunosuppressive therapy and blood pressure control, respectively, resulting in earlier onset of hemodialysis.^20^ Furthermore, a study with black people in South Africa showed that the presence of HRG was associated with an 89-time higher risk for developing HIVAN.^21^ It can be noted that the nephropathies associated with genetic variants of *APOL1* are generally diseases without the involvement of extrarenal organs, which suggests no systemic effects, as will be discussed later in this review.^22^ This variable risk across different etiologies of kidney diseases associated with the HRG suggests that their presence alone is not enough to induce renal injury, but likely requires another factor or a "second hit" to induce kidney disease.^23^ Despite these different diseases being classified according to glomerular pathology findings, they have pronounced interstitial and vascular changes that can be linked to *APOL1* variants.^24^

**Table 1:** List of APOL1 odds ratio studies

Study	Year	Author	Population	OR
Case-control	2010	Genovese(87)	FSGS1(USA)	10.5
Case-control	2010	Genovese(87)	ESRD-HTN (USA)	7.3
Case-control	2010	Tzur(15)	ESRD-HTN2	4.9
Case-control	2011	Kopp(17)	HIVAN1 (USA)	29.2
Case-control	2011	Kopp(17)	FSGS1 (USA)	16.9
Case-control	2011	Papeta(88)	FSGS1 (USA)	3
Case-control	2011	Papeta(88)	HIVAN1 (USA)	3
Populational	2011	Friedman(29)	Dallas Heart Study Malb3 (USA)	3
Populational	2011	Friedman(29)	Dallas Heart Study CKD1 (USA)	4
Case-control	2012	Fine(89)	HIV1 (USA)	3
Coorte ESRD	2012	Lipkowitz(90)	AASK CKD1(USA)	4
Coorte ESRD	2012	Lipkowitz(90)	AASK proteinuria1 (USA)	6
Coorte ESRD	2012	Lipkowitz(90)	AASK1 (USA)	3
Case-control	2013	Ulasi(91)	CKD1 (Nigeria)	6
Coorte ESRD	2013	Parsa(26)	AASK progressionESRD1 (USA)	2
Coorte ESRD	2013	Parsa(26)	CRIC progression CKD4 (USA)	3
Populational	2013	Foster(92)	ARIC - CKD5 (USA)	2
Populational	2013	Foster(92)	ARIC - ESRD1 (USA)	3

1 = only African descendants with CKD 2 = African descendants and Hispanics with CKD 3 = general population 4 = general population with CKD 5 = general African descendants

More recently, Hoy *et al*.^20^ examined microscopy sections of healthy kidneys from AA and described the phenotypes according to their *APOL1* profile. The study showed that individuals with the HRG lose glomeruli since the first decades of life, while the general healthy population begins to lose glomeruli from the age of fifty. These differences persisted after adjusting for variables such as hypertension, which is present at higher incidence in carriers of the HRG. The glomeruli loss in the HRG group reached 350,000 nephrons in the first 38 years, a significant number considering that a normal kidney contains about 900,000 nephrons. In addition to the reduction in the total number of glomeruli, there was an increase in the glomeruli volume. This can be explained by a compensatory hypertrophy, resulting from the inverse relationship between the number and volume of glomeruli.^20^

The early renal function decline appears to occur in a similar manner in diabetic nephropathy, with manifestation of albuminuria followed by rapid deterioration of renal function.^25^ This suggests that the presence of proteinuria may be a screening indicator to the presence of HRG, since in the absence of proteinuria the chance of deterioration in renal function is considered to be small. This would be interesting from an economic point of view, since the test for detection of variants is still expensive. In contrast, another study showed that even in patients without proteinuria the presence of HRG was a risk factor for progression of CKD.^26^ Analyzing AA participants from the NEPTUNE study (Nephrotic Syndrome Study Network), it was observed that independently of renal disease (minimal lesions disease, FSGS, membranous nephropathy), those with the HRG were associated with decreased glomerular filtration rate and reduced chance of complete remission with treatment, even after correcting for multiple variables. This means that although the HRG does not increases the occurrence of certain diseases, its presence dictates a worse prognosis.^27^ The association between non-diabetic forms of CKD and the presence of *APOL1* HRG is so significant that a reclassification of the causes of CKD has already been proposed, in order to group the various kidney diseases associated with *APOL1* variants.^28^

Regarding diabetic nephropathy, the most common cause of CKD, studies show little or no association between the presence of the HRG and its development.^29^ Once established, however, diabetic nephropathy progresses faster in patients with the HRG.^26^ Yet, this has also been observed in AA without the HRG. Therefore, the difference in diabetic nephropathy progression between ethnic groups may in part be due to traditional risk factors and socioeconomic differences^26^ in addition to yet to be discovered risk factors and genetic factors.

## ORIGIN OF MUTATIONS

The European population distanced itself from the African population approximately 40 thousand years ago and since then various differences in genetic variants have emerged. These differences are explained by genetic selection forces that have taken place over the millennia.^30^ It is estimated that *G1* and *G2* variants have arisen about 10 thousand years ago, thus after the separation of the European and African strains.^31^ The variants originated in the sub-Saharan Africa and were brought to the American continents trough slave trade. It is estimated that between the sixteenth and the nineteenth century about twelve million African slaves were brought to the Americas, mainly from sub-Saharan west coast and Southeast Africa, regions with high prevalence of the *APOL1* HRG.^16^^,^ 32 Since the absence of one does not discard the other, it is important to genotype for the two variants.^16^ The *G2* variant appears to be the oldest and is widely found in sub-Saharan Africa. Its prevalence in the region is around 10%^16^, while the variant *G1* appears to have undergone more recent selection, having less uniform prevalence.

## PREVALENCE STUDIES

**Figure 1:**
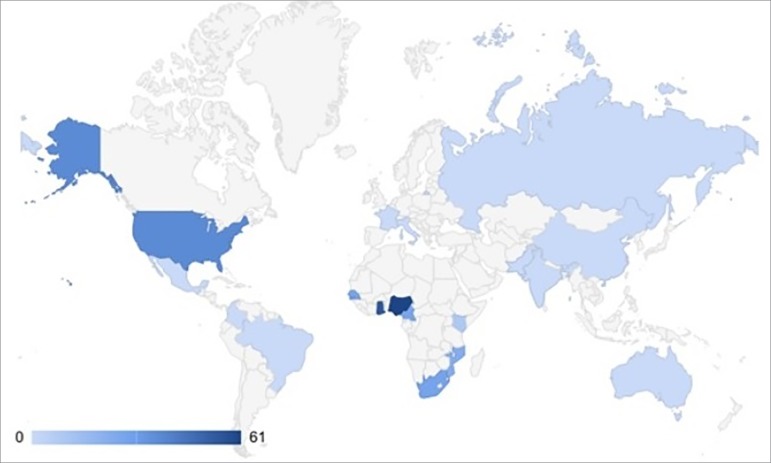
World prevalence of the risk variant (at least one alelle).

The prevalence of HRG varies greatly between countries (Table 2, Figure 1). In West African countries such as Nigeria, where the mutations originated, the prevalence of at least one variant allele is as high as 45%, while in a group of South Africans with HIV, this number reaches a staggering 79%.^21^ AA living in the United States, one of the destination countries of intercontinental slave traffic, also have a high prevalence of HRG. Studies have shown that the *G1* allele is found in 20 to 22% of this population and the *G2* allele in 13 to 15%, while 10 to 15% have two alleles. Other countries, such as Australia^33^ and India^34^, on the other hand, have studies showing absence of the HRG in their populations.

**Table 2:** World prevalence of the risk variants^1^

Country	Prevalence (%)	Country	Prevalence (%)
Nigeria	61	France	0
Ghana	54	Colombia	0
USA	37	Mexico	0
South Africa	28	Pakistan	0
Senegal	27	Australia	0
Cameroon	27	India	0
Mozambique	23	China	0
Congo	16	Italy	0
Central African Republic	15	England	0
Kenya	9	Russia	0
Brazil	0	Israel	0

1 at least one risk allele

Source: J Am Soc Nephrol 2011;22(11):2129-37; Kidney Int 2016;90(4):906-7; Kidney Int 2017;91(4):990.

As far as we know, in Brazil, only two studies were published on the subject. In the first, using data from the International HapMap Project, Kopp et al. analyzed 49 individuals from 2 indigenous tribes (Karitiana and Surui) and found no mutation in any of the participants.^17^ In the second, Colares et al. analyzed 196 patients with lupus nephritis (a condition knowingly associated with HRG), investigating whether certain genetic polymorphisms have a statistically significant association with the disease. They found, however, no relation to the risk alleles G1 and G2, perhaps because less than half of the participants were African descendants.^35^ There is an ongoing prevalence study of the HRG in a sample of African descendants in chronic hemodialysis in Brazil from our group.

Some authors suggest that there are genetic differences between the American and Brazilian Afro-descendant populations, since the former would be much more homogeneous, with up to 80% of the genome originated in West Africa.^92^ Genetic studies showed a strong presence of genetic variants only in the sub-Saharan African regions, mainly on the West coast (and not in the North, South, and East). Analyzing historical accounts, it can be seen that most of the slaves brought to the US came from Senegal, Gambia, Nigeria, and Cameroon^93^, just off the West African sub-Saharan coast. This would increase the chance of finding homo or heterozygous individuals for the mutations under study. Brazil, on the other hand, would have a distinct geographical origin of the migratory flow of slaves, which would reduce the possibility of finding individuals with the mutation in this population. Studies of the ancestry of Afro-descendants in Brazil, however, have recently determined that the greatest genetic load of this population came from Cameroon, Ghana, and Senegal, also regions with high prevalence of variants^94^. That is, the theory that other subgroups of Afro-descendants would have come to Brazil does not hold, since both countries received slaves from the same places of origin, precisely areas of high prevalence of variants of risk. What happened in Brazil was a greater miscegenation among Afro-descendants with Indians and whites, (unlike in the USA, where racial segregation has always been very strong), contributing to a greater genetic variety in this population.

## PHYSIOLOGY

The selective pressure suffered by the variants likely occurred due to a protective effect against a subspecies of *Trypanosoma brucei*.^13^ This parasite causes trypanosomiasis, an endemic disease in Africa also known as sleeping sickness.^17^ To better understand this mechanism, we must remember that *G1* and *G2* are genetic variations in a gene called *APOL1* (*G0* being the wild type variant), present only in humans and certain primates. The *APOL1* gene is a member of a family of six genes (*APOL1, APOL2, APOL3, APOL4, APOL5* and *APOL6*), clustered on chromosome 22.^36^ The APOL1 gene encodes a protein called apolipoprotein L1 (apol1), which is present in the circulation at a concentration of approximately 0.3 mg/dL.^36^ The apol1 particle circulates in a specialized high-density lipoprotein (HDL), along with the haptoglobin-related protein, which acts as a receptor for entry into the trypanosome. It acts by suppressing the replication and spread of the parasite in the body, thereby avoiding chronic trypanosomiasis.^37^^,^ 38 Hence, humans have acquired specific immunity against *Trypanosoma brucei brucei* trough this protein. The apol1-HDL particle also circulates connected to the IgM molecule.^23^

The protein expression of apol1 occurs in organs such as liver, pancreas, and kidney, as well as various types of cells, such as mononuclear phagocytes, placental cells, neurons in the prefrontal cortex and endothelial cells.^36^^,^ 39 In the kidney, apol1 was found to be expressed in endothelial cells, on the epithelium of the proximal convoluted tubule, podocytes, renal arteries, and renal arterioles.^40^ This finding may explain the pattern of glomerulosclerosis plus a component of vascular involvement and interstitial fibrosis.^41^ It has been observed in biopsies that, in addition to vascular and interstitial fibrosis, patients with the HRG have significantly more tubular atrophy.^27^ In contrast, in tissue culture experiments, podocytes expressing apol1 show no signs of toxicity.^41^

Trypanosomes are flagellated protozoan parasites that replicate in the bloodstream of several mammalian species, following inoculation by means of an infected mosquito bite from the species *Gloossina* (also known as tsetse flies). There are three main species of *Trypanosoma: Trypanosoma brucei brucei, Trypanosoma brucei rhodesiense* and *Trypanosoma brucei gambiense*. The *Trypanosoma brucei brucei* is unable to infect humans because of the tripanolytic effect of apol1 (wild type), present in human blood. Apol1 acts by forming an anionic pore in the liposomal membrane of the parasite, the increase in anion permeability results in cell swelling followed by death.^42^ The protective effect by Apol1 is also found in other primates such as gorillas and baboons. The tripanolytic action of apol1 was already known before the discovery of the HRG and has been widely studied.

The *Trypanosoma rhodesiense* occurs in eastern Africa and is responsible for the acute form of the disease. Conversely, the *Trypanosoma gambiense* occurs in west and central Africa, causing the chronic form of the disease, which is the most common and can be fatal.^16^^,^ 43 ^,^ 44 The *Trypanosoma rhodesiense* developed a mechanism to escape cell death caused by the tripanolytic factor, thus reclaiming the ability to infect humans and primates and consequently cause sleeping sickness.^45^ This mechanism involves the expression of a factor called serum resistance-associated (SRA) protein, which binds to the C-terminal portion of apol1 (gene region where the G1 and G2 mutations are located), counteracting the tripanolytic action of apol1.^46^ The structure of apol1 is comprised of five regions: secretory (which allows expression of serum protein), pore forming, membrane addressing, leucine zipper region, and the region that interacts with the SRA at the c-terminus. The three regions responsible for the tripanolytic activity are the pore forming, the membrane addressing (pH-sensitive), and the region that interacts with the SRA.^16^

The risk variants are located in the C-terminal portion and the resulting amino acid changes prevent SRA to bind and neutralize the action of the tripanolytic factor.^46^ By neutralizing the SRA, apol1 can again exert its tripanolytic function, which is a significant evolutionary advantage in endemic regions of trypanosomiasis and thus may explain the high prevalence of *G1* and *G2* mutations in these regions of Africa.^13^ The *G2* variant is more potent against T*rypanosoma rhodesiense* and older than the *G1* variant.^16^ The high prevalence of *APOL1* variants that are resistant to SRA in West Africa, and therefore can kill *Trypanosoma rhodesiense*, and the virtual absence of variants in East Africa, where *Trypanosoma rhodesiense* is widespread, allows us to conclude that *Trypanosoma rhodesiense* has been eradicated in regions where the variants are present, since they avoid the connection of the SRA to apol1.^47^ The elimination of *Trypanosoma rhodesiense* in West Africa probably left room for the expansion of another species, the *Trypanosoma gambiense*, which resists apol1 by a different mechanism and is therefore immune to the effect of *G1* and *G2* variants. Since the variants emerged about 10,000 years ago, a relatively short time when it comes to frequency of alleles in populations, it is possible that its spread to East Africa is not yet complete.^47^ In short, the evolutionary race of host (human) versus pathogen (*Trypanosoma*) resulted in the occurrence of the *G1* and *G2* mutations, which provided evolutionary advantage to its carriers. The advantage, however, came at a cost; it resulted in an increased risk of developing CKD, as discussed below.

## APOL1 TOXICITY

The immune function of the risk variants occurs in a dominant fashion, i.e., the presence of only one allele (heterozygous form, either *G0/G1* or *G0/G2*) is sufficient to confer protection against *Trypanosoma rhodesiense*. As for the development of CKD, it is necessary to have the presence of two alleles in homozygous (*G1/G1* or *G2/G2*) or compound heterozygote (*G1/G2*) form.^23^ This is analogous to the correlation between sickle cell anemia and protection against malaria, another African endemic disease: while heterozygous and homozygous for the mutation in hemoglobin are protected against malaria, only homozygotes develop sickle cell anemia.^48^

This recessive pattern allows two possible conclusions: the presence of *G0* protects against CKD (by loss of function of the protein having the mutation) or *G1* and *G2* variants cause CKD (gain of function).^49^ Against the theory of protection provided by the wild variant (*G0*) is the fact that the *APOL1* gene does not appear to be essential for the functioning of the kidney, since a human homozygous null genotype for this gene have been identified and apparently retains normal renal function after several years of follow up.^50^ Furthermore, the *APOL1* gene is not present on every mammalian species, only in certain primates. Even the primate with the greatest degree of genetic similarity to the human species, the chimpanzee, lacks a functional *APOL1* allele^36^, suggesting that this gene is not essential in mammals.^51^ The second hypothesis, gain of function, is now the most widely accepted because there is mounting evidence of cytotoxic effects of HRG in several models surveyed.^23^ Understanding the action of the *APOL1* variants is hampered by this gene's absence in established animal species used for models of kidney disease (i.e., mice models) and the fact that apol1 is toxic in cultured cells (i.e., HEK293 cells), when present in supraphysiological concentrations, by decreased cell viability.^52^^,^ 53

The development of severe forms of CKD in adulthood does not appear to have been a factor strong enough to counter the selective pressure of having resistance to sleeping sickness. This is likely explained by the fact that CKD manifests at an older age, when the reproductive period of the individual has already passed, exerting no significant natural selection disadvantage.^47^ Both variants have an unusual combination of high prevalence and strong effect, resultant of selective pressure related to innate immunity.^54^ The *APOL1* variants are an unusual example where the mutation in a single gene is associated with increased risk of a complex disease.^24^

## "SECOND HIT", ANOTHER FACTOR REQUIRED FOR CKD DEVELOPMENT

Most individuals with HRG do not develop kidney disease without the presence of an additional stress factor or "second hit". The variants are therefore necessary, but insufficient to trigger kidney disease by themselves. The reason for this incomplete penetrance are not yet known and is not currently possible to predict which carriers of the HRG will develop CKD. There seems to be a connection between the stimulus of the innate immunity caused by infections and the apol1 expression in podocytes.^51^ Chronic diseases that cause a persistent inflammatory state and subsequent high production of interferon, such as systemic lupus erythematosus (SLE), have also been associated with *APOL1* HRG.^51^ An increase in the expression of the UBD gene, responsible for the production of proteins involved in the degradation of cellular proteins, was also seen in the HRG carriers, through a mechanism independent of ubiquitin.^55^ This gene has its activity increased through high levels of IFN-gamma and TNF-alpha. With this, the HRG could serve as inducer of a pro-apoptotic state and could link an exaggerated immune response in *APOL1* HRG patients with renal damage.^55^

## EFFECT OF APOL1 IN PODOCYTES

Apol1, being the only protein from the apolipoprotein family present in the blood, gives it unique features, such as protection against SRA. But its general function is probably linked to the function of other apolipoproteins, which have structural and functional similarities to the BCL2 family of proteins. This family of proteins participates in the control of apoptosis and cell autophagy.^56^ In rats, the downregulation of autophagy in the kidney leads to senile sclerosis of podocytes, which is similar to what occurs in humans with HRG.^57^ It is tempting thus to propose that the HRG interferes with podocyte autophagy, leading to progressive glomerulosclerosis. Recent studies have shown that humans also have age-related podocyte density reduction. In young individuals, the normal density is above 300 per 10^6^ µm^3^, dropping to less than 100 per 10^6^ µm^3^ in individuals over seventy years. This translates to an average of 0.9% reduction in annual density, similar to the annual decline in renal function, which is 0.8% per year after thirty years of age.^58^ This explains why end stage chronic renal disease (ESRD) increases exponentially with age, and why age is the greatest risk factor for loss of kidney function.^59^

To better understand the effects of the HRG in the kidney, genetically modified mice expressing *APOL1 G2* (two amino acid deletion) were created. It was observed that mice with the mutation had podocytes with lower density.^49^ This represents a major challenge for a specialized cell such as the podocyte, responsible for maintaining all glomerular filtration surface covered with its foot processes, inevitably causing stress on the entire system. This data supports the hypothesis that AA individuals with HRG have an inadequate number of podocytes for their age. Lower podocytes density can represent a risk factor predisposing individuals to glomerular diseases, since a kidney would further reduce the podocyte reserve, leading to ESRD. Following this model, the survival of glomeruli in sufficient numbers throughout life will depend on the total cumulative loss of podocytes, the increased glomerular volume and the ability of the remaining podocytes to adapt to the stress resulting from the fall of podocyte density.^59^ Another study in mice was performed recently by Beckerman et al.^60^, in which he showed that kidney disease (characterized by albuminuria, azotemia, glomerulosclerosis, and podocyte foot-process effacement) was caused by podocyte-specific expression of the HRG (but not by the G0 allele). This study also suggests that the effects of the HRG are podocyte-specific (rather than nonspecific toxicity), by showing that the expression of the HRG in kidney tubules did not result in kidney disease. A recent study made by Lan et al. in human podocytes showed that the HRG is associated with increased podocyte necrosis, by failure in the permeability of lysosomal membranes.^39^ Observational studies have also highlighted the relationship between decreased podocyte density and diseases such as diabetic nephropathy, IgA nephropathy, hypertensive nephrosclerosis and transplant glomerulopathy.^59^ Human models have shown that once the loss in the density of the podocytes is above 40%, glomerular disease becomes progressive and irreversible, regardless of cause, resulting from the stimulus of cell hypertrophy.^61^ It is clear that there is a direct relationship between reduction in podocyte density (volume/number) and development of glomerulosclerosis.^62^

Three mechanisms have been proposed to explain this reduction in density. The first is the hypertrophy of the glomeruli, which makes the same number of podocytes be responsible for a greater filtration surface and thus generate a stress that forces the podocytes to divide. Morphological studies in healthy AA with the HRG showed increased glomerular hypertrophy related to age, which may predispose to kidney disease.^20^ The requirement of podocytes to divide may lead to changes in the actin cytoskeleton, which prevents them from maintaining its physiological function. This results in disruption of the podocytes' function, glomerular tuft collapse, and consequently FSGS.^63^ The second mechanism is the reduction in the absolute number of podocytes by death or cell disruption. It has been shown that humans and rodents podocytes can be detached from the glomerulus without dying.^64^ Finally, podocytes dysfunction can occur when they fail to perform their physiologic role.^59^

## EFFECT OF CIRCULATING APOLIPOPROTEIN L1

Studies have shown that AA have a higher serum level of apol1 compared with other ethnic groups, which may reflect an increased production or a decreased clearance/degradation of the protein. In addition, it has been shown that apol1 present in the kidney can be derived either from endogenous synthesis or extracellular sources, in both healthy and diseased kidneys.^41^ If high circulating levels of apol1 were related to higher risk of developing CKD, its measurement could be used as a biomarker to discriminate high-risk patients among carriers of the HRG.^23^ Researchers then suggested that the toxicity of genetic variants could be more dependent on the circulating versus the renal expressed apol1.^23^ Arguments in favor of this hypothesis include the fact that apol1 protein is connected to HDL molecules, which are partly filtered by the kidneys (being even captured by podocytes) and that IgM molecules (which also carry apol1) may contribute to the pathophysiology of some glomerular diseases. A recent study, however, showed no relationship between serum levels of apol1 and development of CKD.^23^ There was also no relationship between circulating levels of apol1 and certain phenotype of the HRG^40^, on the contrary, studies suggest that renal expression of apol1 is more important for the renal pathogenesis than the circulating apol1. Ojo et al. showed that survival of a kidney graft is abbreviated in patients with transplant kidneys from HRG donors, independent of the *APOL1* status of the recipient.^65^ In conclusion, the data described above supports the hypothesis that the circulating apol1 is not involved in renal pathogenesis.

## APOL1 AND KIDNEY TRANSPLANTATION

It is known that living donor kidneys have longer survival than those from deceased donors. This occurs by a series of factors: better preoperative evaluation of renal function and anatomy; elective surgeries (in optimized working conditions); reduced cold ischemia time (no delay in the definition of the allocation of the graft); and generally better antigenic compatibility. There is also the use of immunosuppressive drugs, which are often nephrotoxic and a deleterious effect common in both types of transplantation.^24^

Studies in humans and animals have shown that blood pressure and salt sensitivity accompany renal allografts and directly impact the recipient's homeostasis.^66^ It is therefore possible to assume that the increase in risk of nephropathy also carries over with the allograft in transplantation. A study of 136 AA deceased donor grafts showed that the only factor affecting the survival of grafts after multivariable adjustment was the presence of HRG. The study also showed that donor ethnicity, regardless of *APOL1* genotype, had no impact on survival. This supports that the worse graft survival from AA donors is due to the presence of the *APOL1* HRG, irrespective of donor race. In addition, histology of most patients with HRG that completely lost graft function was consistent with diseases associated with *APOL1* HRG.^67^ A subsequent study, evaluating 675 AA donor allografts, also showed that the presence of HRG was an independent risk factor for worse transplant kidney outcome.^68^ Another study showed that AA recipients have higher allograft survival when the donor is not AA.^24^ Transplanted kidneys with HRG are at risk for early loss of renal function, but studies have shown that those that continue to function for over 3 years tend to have an average allograft lifespan.^24^ Reeves-Daniel et al. showed that grafts derived from carriers of HRG have twenty months less survival.^67^ This led to the suggestion of genotyping AA donors for better evaluation of the potential graft survival. Howeve, there is still no consensus on the subject, because the mechanism of early loss in these grafts is still unknown. Could more frequent monitoring of HRG allograft lead to improved graft survival and justify the increase in costs with genetic testing? Moreover, would knowing the allograft genotype play a role in the decision of organ allocation by scoring it as lower quality? Would these measures ultimately improve patient care and kidney outcomes? These questions need to be further debated by the scientific community, since a premature decision to use the data could lead to longer transplant waiting time and lower rates of living donation, as already occurs in the AA population.

To assess the impact of the recipient *APOL1* genotype on renal graft survival, Lee et al. studied 119 AA receptors and showed that although 49% had HRG, after five years there was no difference in renal function between the HRG versus low risk genotype groups. This suggests that the receptor *APOL1* genotype does not influence graft survival.^69^ One comes to the conclusion that there is a well-established worse survival of grafts derived from AA donors and that this phenomenon is independent of receptor ethnicity.^65^

An important issue not yet answered is the impact of *APOL1* genotype after nephrectomy in living donors. It is known that 0.10% of Caucasians and 0.51% of AA living donors progress to ESRD over the years^70^ and it is assumed that this increased risk of worse outcome in AA reflects the higher CKD risk this ethnic group has versus the general population.^71^ One possibility would be to perform *APOL1* genotyping in AA being considered for kidney donation. Other authors suggest extreme caution in the upward vertical donation (children to parents) in AA, as these children may have kidney disease not yet manifested. The fact is that more clinical studies are needed to bring clarity to this matter. Interestingly, if shown that the genotype of *APOL1* is the factor responsible for worse survival of grafts derived from AA (and not the race itself), the pre-transplant risk assessment formulas would have to be reformulated.^24^ In relation to deceased donors, it has been suggested that *APOL1* genotyping be tested along the routine deceased kidney donor panel. The use of grafts with the presence of HRG would be indicated in situations where donors with expanded criteria are suitable.^24^ These potential screening modifications should be taken with caution since causality of *APOL1* HRG has not been established, only a strong association with disease. Recommending HRG AA carriers not to donate without strong evidence of causality would decrease even further the number of living donations across AA individuals. Moreover, living kidney donor relationship in AA and Caucasian populations are different. One study assessing 1000 donors and recipient records showed that AA are less likely to receive a kidney from an unrelated person (17% vs 37%, *p* = 0.001) and less likely to receive spousal donations (6% vs. 13%, *p* = 0.001). In contrast, donation from child to parent is more common in AA (33% vs. 15%, *p* = 0.0001).^72^

Studies show that around 30% of cases of FSGS have recurrence after renal transplantation (percentage that reaches 86% in children), with a generally very unfavorable evolution. Genetic forms (including those related to the *APOL1* gene) do not appear to be in the high-risk group for this unfavorable outcome. The soluble urokinase receptor (suPAR), on the other hand, has been recently found in high levels in the urine of transplanted patients with recurrence of the disease, and may serve as a potential marker in the future.^95^

## APOL1 AND CARDIOVASCULAR RISK

There is a well established increase in cardiovascular risk in patients with CKD, even in the early stages of the disease^73^, but the explanation for this increased risk is still uncertain. There is evidence that the cardiovascular risk is also higher in the AA population in general^74^, which could be explained by socioeconomic factors. On the other hand, AA patients with ESRD have reduced cardiovascular risk^75^, which can be explained by the survival effect, as the high risk of mortality in earlier stages of CKD ends censoring those with higher cardiovascular risk. Because of the known expression of apol1 in extrarenal tissues such as vascular tissue, the presence of HRG could be a new risk factor for cardiovascular disease.

The first study to address this issue found no association between the presence of HRG and increased mortality, but this study had low statistical power to draw conclusions, due to the low mortality rate of the participants.^26^ In contrast, the Jackson Heart Study, using the same recessive model of the *APOL1* variants, showed a two-fold increase in risk of cardiovascular events in subjects with the presence of the HRG. The same risk increase was found in the study using the population of the Women's Health Initiative. Interestingly, in this study there was no association between the HRG and cardiovascular events precursors, such as left ventricular hypertrophy, besides a paradoxical decrease in coronary calcification.^76^ A possible explanation of this increased cardiovascular risk would be a dysfunction in the apol1-HDL particle.

Two new studies addressing the issue came to different conclusions. The first was with data of the SPRINT study (Systolic Blood Pressure Intervention Trial), showing no relationship between *APOL1* genotype and history of coronary revascularization, carotid revascularization or acute myocardial infarction. This study only confirmed the relationship of HRG with albuminuria and CKD.^77^ The second study did analysis of data from the AA-DHS study (African-American Diabetes Heart Study) and showed that the HRG are associated with surprising reduction in the risk of death.^78^ These opposing conclusions regarding the initial studies may be explained by different methodologies, but show that more studies are needed on the subject. Also offering evidence against the association of *APOL1* HRG and cardiovascular risk are numerous genomic studies and studies with admixture linkage mapping that did not identify the chromosome 22 (where the *APOL1* gene is located) as a locus for blood pressure control mechanisms. This suggests that HRG probably does not contribute to primary hypertension.^20^

It will be important to determine a possible association between the presence of HRG with increased or decreased cardiovascular risk as this will influence the clinical management. If the *APOL1* HRG contributes to risk, the same treatment used to slow the progression of CKD may also help in preventive measures against cardiovascular events. It is clear the need for further studies directed to this subject, as well as for better understanding of the mechanisms of action of *APOL1*.

## APOL1 AND HIVAN

In 2013 there were around 35 million people infected with the human immunodeficiency virus (HIV) in the world, with almost 25 million people in sub-Saharan Africa alone. Since the beginning of the HIV epidemic, nearly forty million people died from the disease. In absolute numbers, it is the second leading cause of mortality in the modern era (since 1900), losing only for the 1918 flu (which caused between 50-100 million deaths). The incidence has decreased in recent years and been concentrated in specific areas, especially in sub-Saharan Africa, accounting for 70% of new cases. Just over 35% of HIV-infected people receive the highly active antiretroviral therapy (HAART) regularly and the treatment rate in sub-Saharan Africa is similar to the rest of the world.^79^

HIVAN was the first kidney disease to be described in an HIV patient (in 1984) and also the first collapsing glomerular disease to be described.^80^ Other kidney diseases associated with HIV include HIV-associated immune-complex, thrombotic microangiopathy, and disorders related to antiretroviral toxicity. The clinical presentation of HIVAN includes proteinuria and renal dysfunction with kidney pathology consistent with collapsing FSGS, microcystic tubular dilation, and interstitial inflammation. Before the advent of the highly active antiretroviral therapy (HAART), in 1995, HIVAN affected between 3-10% of those infected with HIV, with preponderance in the AA population. Without HAART, HIVAN usually advanced rapidly to ESRD.^79^ In the US, after the introduction of HAART, there was a 60% reduction in the risk of progression to ESRD in patients with HIVAN.^79^ A French study even showed a change in the prevalence of nephropathy after the introduction of HAART, from collapsing FSGS to non-collapsing FSGS.^81^ On the other hand, drug related nephrotoxicity is an increasingly serious problem, which may occur even in patients with previously normal function.^82^

Since the beginning, it was clear that AA patients were more affected with HIVAN than patients of other ethnicities. It is now known that this genetic predisposition is due to the association with G1 and G2 mutations in the APOL1 gene.^13^ This relationship between HIVAN and HRG is so direct that countries such as Ethiopia, where the variations are practically not found, have very low prevalence of HIVAN.^83^ In patients with HIV in sub-Saharan Africa, HIVAN is the most common kidney disease and also the leading cause of mortality in patients without antiretroviral treatment.^84^ Studies show that without treatment, 50% of seropositive AA with HRG develop HIVAN^17^, making HIV the environmental factor with the greatest interaction with the HRG. One possible explanation is that HIV induces interferon production, hence boosting the expression of the gene and subsequent increasing apol1 production from the variants.^39^ To demonstrate this relationship, a study showed that AA patients carriers of the HRG undergoing treatment with interferon developed collapsing FSGS.^51^ It was also observed that the Toll-like receptor 3 (TLR3) molecule, which functions as an interferon agonist, increases the expression of apol1 in podocytes and endothelial cells^51^. An issue to be clarified is why 10% of AA patients with HIVAN have no risk allele and 20% carry only one allele.^17^ Further research should also focus on factors that protect HIV-infected individuals with HRG from developing HIVAN.

Another open question is how the HRG increases the frequency of collapsing FSGS, since this disease is known for its characteristic of proliferative lesions and increased glomerular cells, while the expected effect of the HRG would be an injury that causes death cell and therefore diminishes the number of cells^85^. Three possible explanations would be:

+ The *APOL1* HRG individuals have increased parietal glomerular cells or other potential predecessors of podocytes, leading to hypertrophy of these strains and interfering with their ability to replace injured podocytes;

+ Paracrine effects occur secondary to the *APOL1* expression in glomerular endothelial cells and podocytes^86^;

+ Th1 cytokines that stimulate secretion of apol1 have an independent effect on endothelial cells and podocytes.

## CONCLUSION

Increasing importance of the HRG is seen in the development of kidney diseases in the Afro-descendant population, being now understood that a myriad of previously disjointed diseases are in fact a spectrum of the same disease. The discovery of HRG, along with other genotypes such as PLA2R (membranous glomerulonephritis), ADAMTS13 (thrombotic thrombocytopenic purpura), and suPAR (focal segmental glomerulosclerosis), suggests that the era of "Precise Medicine" has also reached Nephrology, with the identification of specific genetic mutations causing diseases with high prevalence. One of the goals of this new phase in Nephrology will be to determine biomarkers to predict more accurately the prognosis of renal function than those currently used, such as creatinine and albuminuria. One example is the lower mortality in patients with HRG undergoing rigorous blood pressure control.^87^ The World Health Organization itself has recently shifted its focus from contagious diseases to diseases with genetic susceptibility, targeting therapies that may prevent environmental triggers on liable individuals.^88^

It is also clear that the generic categorization of an individual within a given race is of less value than the determination of their genetic ancestry^89^, as shown in kidney transplant studies. In this area, there is also the expectation of an increasingly better allocation and estimate organ survival.^90^ It is important, however, to stress that routine screening in black patients with CKD is still not recommended because there is no effective treatment for this group of patients.^91^ Nevertheless, more attention should be paid to the low survival rates of kidneys from donors with HRG.

An area with great potential for research on the subject are the therapeutic possibilities for those with genetic variants. One of them was described by Beckerman et al., who showed that there was a decrease in HRG-induced cytotoxicity with specific caspase 1 inhibitors. They concluded that other inhibitors of interleukin-1 or inhibitors of cellular death may also function in a similar way. Further research is needed in this area to determine not only the best clinical utility for HRG screening, but also to provide a therapeutic approach. The key in this process will be the understanding of which second hits trigger kidney diseases in patients, since only a minority of them develop nephropathies.
